# An integrated full-head OPM-MEG system based on 128 zero-field sensors

**DOI:** 10.3389/fnins.2023.1190310

**Published:** 2023-06-14

**Authors:** Orang Alem, K. Jeramy Hughes, Isabelle Buard, Teresa P. Cheung, Tyler Maydew, Andreas Griesshammer, Kendall Holloway, Aaron Park, Vanessa Lechuga, Collin Coolidge, Marja Gerginov, Erik Quigg, Alexander Seames, Eugene Kronberg, Peter Teale, Svenja Knappe

**Affiliations:** ^1^FieldLine Medical, Boulder, CO, United States; ^2^Paul M. Rady Department of Mechanical Engineering, University of Colorado Boulder, Boulder, CO, United States; ^3^FieldLine Industries, Boulder, CO, United States; ^4^Anschutz Medical Campus, University of Colorado Denver, Denver, CO, United States; ^5^School of Engineering, Simon Fraser University, Burnaby, BC, Canada; ^6^Surrey Memorial Hospital, Fraser Health Authority, Surrey, BC, Canada

**Keywords:** optically-pumped magnetometer, HEDscan, magnetoencephalography, system integration, cross-validation, OPM

## Abstract

Compact optically-pumped magnetometers (OPMs) are now commercially available with noise floors reaching 10 fT/Hz^1/2^. However, to be used effectively for magnetoencephalography (MEG), dense arrays of these sensors are required to operate as an integrated turn-key system. In this study, we present the HEDscan, a 128-sensor OPM MEG system by FieldLine Medical, and evaluate its sensor performance with regard to bandwidth, linearity, and crosstalk. We report results from cross-validation studies with conventional cryogenic MEG, the Magnes 3,600 WH Biomagnetometer by 4-D Neuroimaging. Our results show high signal amplitudes captured by the OPM-MEG system during a standard auditory paradigm, where short tones at 1000 Hz were presented to the left ear of six healthy adult volunteers. We validate these findings through an event-related beamformer analysis, which is in line with existing literature results.

## 1. Introduction

The first optically-pumped magnetometers (OPMs) were developed in the 1970s (Livanov, 1977) and have long been reaching sensitivities in the low picoTesla range. More recent innovations such as the spin-exchange relaxation-free (SERF) magnetometer (Happer and Tang, 1973; Allred et al., 2002), the integration of vertical-cavity-surface emitting lasers (VCSELs) (Affolderbach et al., 2000), the single-beam scheme (Dupont-Roc et al., 1969; Shah et al., 2007), and the utilization of microfabrication techniques (Liew et al., 2004; Schwindt et al., 2004), have led to highly sensitive OPMs in small packages and enable practical multi-channel arrays (Alem et al., 2017; Borna et al., 2017; Knappe et al., 2019). Early cross-validation studies comparing OPMs to superconducting quantum interference device (SQUID) recordings of biomagnetic signals (Johnson et al., 2010; Knappe et al., 2010; Wyllie et al., 2012), were critical in building confidence in OPM technology. The first commercial standalone sensors have enabled users to make biomagnetic recordings with single or small numbers of sensors (Shah and Wakai, 2013; Boto et al., 2017).

On-scalp sensors for MEG have been studied in several simulation studies, revealing their advanced performance compared to cryogenic systems. These studies have found that on-scalp sensors like OPMs can improve localization accuracies, particularly in small children who face disadvantages in large static cryogenic helmets due to the distance between sensors and the scalp (Luessi et al., 2014). One study predicted a fivefold improvement in sensitivity for adults and a clear improvement in spatial resolution and reconstruction accuracy (Boto et al., 2016). Another theoretical study concluded that an OPM MEG system can yield 7.5 times higher signal power and that the minimum norm-based point spread functions were on average 2.4 times more spread for a cryogenic MEG (Iivanainen et al., 2017). Finally, higher information content has been predicted for on-scalp sensors (Riaz et al., 2017).

Despite these promising predictions, technical challenges have made it difficult to realize the theoretical advantages in experiments. Several sensory-induced experiments have recorded very high signal amplitudes (Borna et al., 2017; Knappe et al., 2019) in agreement with the predictions, but these factors such as the availability of large arrays of OPM sensors, the exact knowledge of the on-scalp sensor positions and orientations, scale-factor drifts, crosstalk, linearity, and response bandwidth, have prevented them from translating into better localization accuracy.

## 2. The dense full-head OPM-MEG system

**Figure 1:**
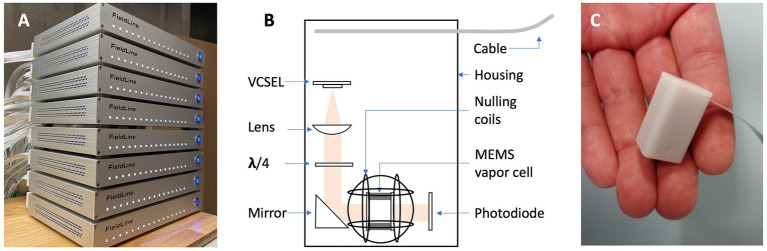
**(A)** Photograph of the integrated controls system for 128 sensors. **(B)** Drawing of the OPM sensor schematic. **(C)** Photograph of an OPM sensor head.

In this study, we demonstrate a 128-channel OPM MEG system that integrates small-sized sensors with a footprint of 13 mm × 15 mm to facilitate high-density recordings. The OPM sensors are connected to an integrated control system (Figure 1A) consisting of 8 electronic chassis. Each chassis can operate up to 16 sensors and can accommodate one stimulus input (a total of 8 inputs for the system). The sensor head is illustrated in a drawing and a photograph in Figures 1B,C, respectively.

The OPM sensors in our system use a VCSEL to emit light at a wavelength of 795 nm that is resonant with the D1 line of Rubidium. The light is collimated and circularly polarized before passing through a microfabricated vapor cell filled with Rubidium and Nitrogen, after which it is detected by a photodiode. To minimize the distance between the vapor cell and the scalp, the center of the vapor cell is 5 mm from the outer surface of the sensor housing. Our OPM sensors operate in the zero-field regime and require a magnetically shielded room (MSR). An integrated set of three orthogonal nulling coils inside the sensor head surround the vapor cell and can compensate for residual magnetic fields of up to ±100 nT. Since the DC magnetic fields in our 4-layer MSR (alternating 3 mm mu-metal and 8 mm aluminum panels, by Lindgren RF Enclosures) reached 150 nT, we used a pair of large panels and wrapped a series of global field coils to further reduce the DC ambient field to below 30 nT at the location of all sensors. The global field coils can generate three orthogonal homogenous magnetic fields and five first-order gradient fields (Tsai et al., 2008). The sensors can record the absolute DC field. The currents in the global field coils were adjusted manually while monitoring the sensor outputs to minimize the field across the array. The currents in the coils remained at constant values during the measurements.

**Figure 2:**
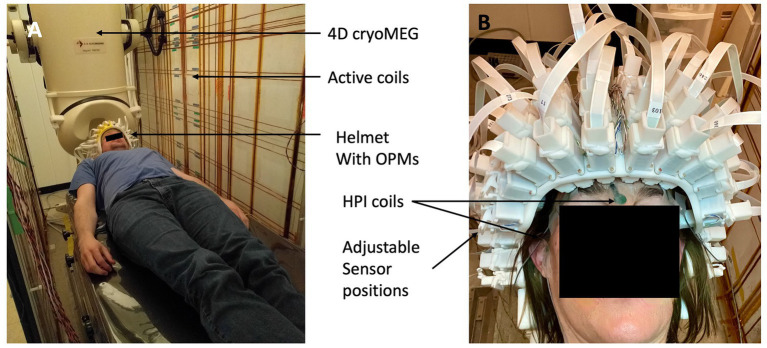
**(A)** Photograph of the setup inside the MSR. The same patient bed was used for both OPM MEG and cryoMEG recordings. The active coil panels can be seen on the side. The dewar of the 4-D Neuroimage cryogenic MEG system can be seen in the background. **(B)** Photograph of the adjustable, self-localizing helmet.

The OPMs were inserted in our adjustable MEG helmet, which was mounted to the bed of the 4-D Neuroimaging cryogenic MEG system. The helmet allows each sensor to move along a single axial direction (towards or away from the head). This adjustment makes it possible to position each sensor to lightly touch the head of the subject, independent of its size and shape. The sensors can automatically self-localize their positions in the helmet (device) coordinate frame by activating 128 reference coils embedded in the helmet. Each coil is surrounding a sensor slot on the helmet to produce a magnetic field in the sensitive direction of the sensor to minimize cross-axis errors (Borna et al., 2022). The sensors measure these fields and the distance between coil and sensor is extracted, constraining the sensor positions in 3 dimensions. The full setup is shown in Figure 2. The 4-D cryoMEG system used for cross-validation can be seen in the background. It contains 248 axial gradiometers with 1.8 cm diameter coils on 5 cm baselines (Magnes 3,600 WH Biomagnetometer, 4-D Neuroimaging, San Diego).

## 3. System performance

### 3.1. Sensor operation

To operate the zero-field OPM sensors, we used an automatic tuning procedure that first locks the laser frequency to the atomic resonance and stabilizes the vapor cell temperature. Then, it nullifies the magnetic field using the on-sensor coils. This nulling procedure is critical because any remaining DC magnetic field can change the OPM’s scale factor and tilt the measurement axis, depending on the amplitude, frequency, and direction of the ambient field (Cohen-Tannoudji et al., 1970; Borna et al., 2022). For example, an uncompensated field of just 1 nT can lead to a cross-axis sensitivity of around 5%. In addition to each sensor’s self-nulling capabilities, more sophisticated methods are being developed that take advantage of multi-sensor information to improve nulling uncertainties (Robinson et al., 2022).

The OPMs use phase-sensitive detection by using the nulling coils to apply a modulated magnetic field to make the sensor sensitive to fields along the long axis of the sensor. Finally, our sensor operate in a closed-loop feedback configuration in their sensitive axis (Lee et al., 2014; Sheng et al., 2017), where the demodulated photodiode signal is fed back to the sensor coils to continually keep the atoms on the zero-crossing of the resonance.

### 3.2. Closed-loop operation

**Figure 3:**
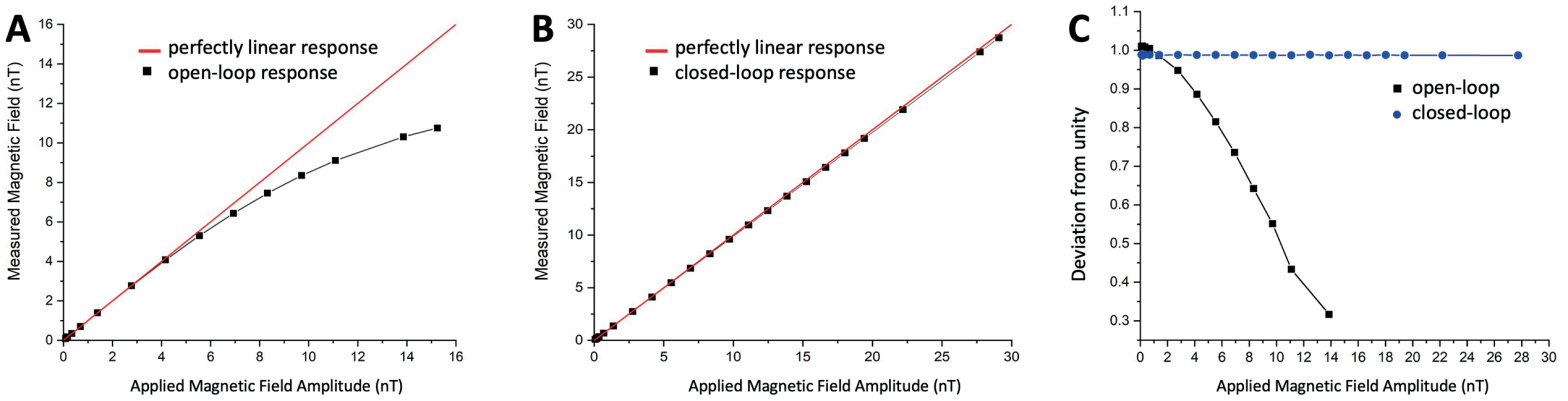
**(A)** Open-loop and **(B)** Closed-loop show the sensor response to a magnetic field at 1 Hz as a function of field amplitude. The black squares are measured field amplitudes and the red line represents 100% linearity. **(C)** Open-loop (black) and closed-loop (blue) sensor linearity shown as a fractional deviation from unity as a function of field amplitude.

The closed-loop operation has several advantages over an open-loop setup. In open loop, the scale factor is determined by the resonance slope of the phase-sensitive signal, which is affected by various sensor parameters such as power, intensity, and wavelength of the light, the degree of polarization, and cell temperature – all of which have to be controlled precisely. Additionally, it is also affected by ambient magnetic field strength and direction, which are difficult to control. Furthermore, the dynamic range of the sensor in open loop is determined by the width of the resonance line, which is only linear within a limited range of 1–2 nT. In contrast, the dynamic range in closed loop is determined by the digital-to-analog converter and gain in the feedback loop. Figures 3A,B demonstrate the linearity of the OPM sensor in open and closed loop, respectively. It was measured by applying an oscillating field at 1 Hz with varying strength and extracting the field amplitude recorded by the OPM sensor. The relative deviation from perfect linearity is shown in Figure 3C. The deviation from linearity of the OPM sensor in open loop is under 0.25% at 1 nT and under 10% at 4 nT, while in closed loop, it remains under 0.25% over the entre dynamic range of 30 nT. It should be noted that the dynamic range of the sensor in open loop was limited to ±7.5 nT and in closed loop to ±15 nT, as shown in Figure 3, in order not to degrade the signal. Since the applied field was bipolar, a field of 15 nT peak-to-peak corresponds to the full dynamic range of ±7.5 nT.

**Figure 4:**
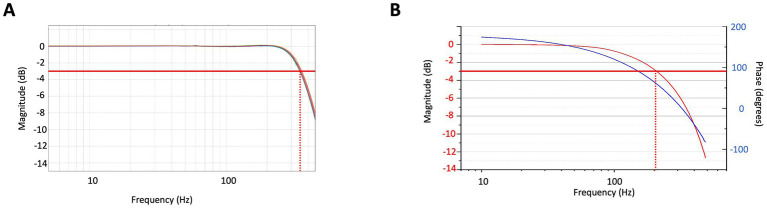
**(A)** Normalized response of the OPM sensors operating in closed loop as a function of frequency shown for 8 sensors. The red line indicates the 3 dB point. **(B)** Normalized response of one OPM sensor operating in open loop as a function of frequency (red) and corresponding phase (blue).

Closed-loop operation not only improves the dynamic range and response linearity of sensors but also increases the response bandwidth of the magnetometer. This is important for achieving unperturbed images with a wide frequency content. Figure 4A depicts the closed-loop response bandwidth of eight sensors, while Figure 4B illustrates the open-loop bandwidth of one sensor for comparison. In closed-loop operation, the 3 dB-response bandwidth is extended to 350 Hz, the gain remains constant to within 0.5% up to 150 Hz and remains unity for all sensors to within 1%. In open loop, for comparison, the 3 dB-response bandwidth is around 200 Hz, but a falloff can already be seen around 30 Hz. In addition, the signal phase is dependent on the frequency over the entire MEG frequency range. When operating large arrays, these characteristics directly influence image quality. Operating in closed loop does have some complications. The system becomes more susceptible to crosstalk between the sensors.

OPM sensors are subject to two types of crosstalk. The first is caused when the modulation field of one sensor is detected by neighboring sensors and is present in both open loop and close loop operation.

The second type of crosstalk is present only when operating in close loop where the feedback field from one sensor is measured by neighboring sensors. The degree of crosstalk depends on the magnetic footprint of the sensor coils outside of the sensor housing. In this case, a coil design with minimal magnetic footprint is essential, when dense sensor arrays are desired. We have implemented a double-coil design (Nardelli et al., 2019), which reduces the crosstalk from the field coils to below 2%, even in the worst case where neighboring OPM sensors are parallel to each other and touching. Since the double-coil design reduces the crosstalk between sensors it can also improve the field nulling of the sensor array in addition to improving the scale-factor stability, stabilizing the effective orientation of the sensors, and reducing phase shifts.

## 4. Measurements with the 128-sensor OPM-MEG system

### 4.1. Empty room data

**Figure 5:**
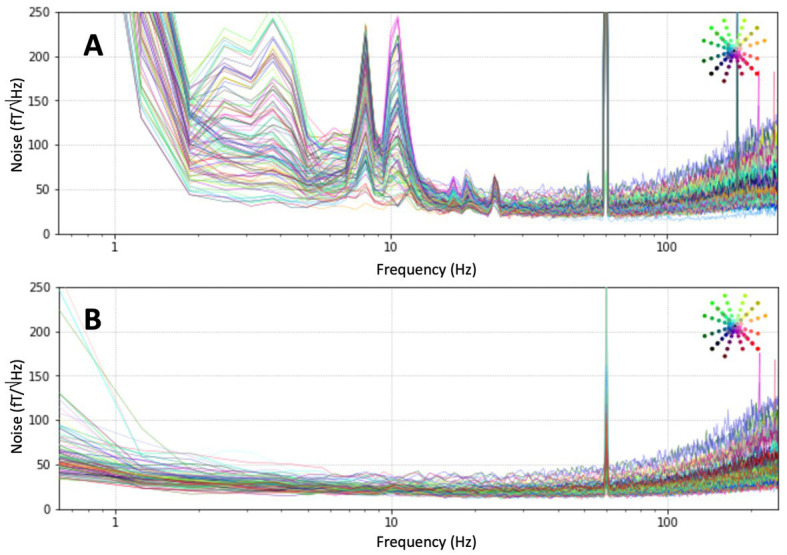
**(A)** Noise floor in the magnetically shielded room. **(B)** Noise floor after signal-space projection (SSP) with 4 projections.

The 128-sensor OPM MEG system was installed inside the shielded room as described above and the DC fields were reduced with the global field coils to below 30 nT over the region of the helmet. The sensors were mounted in the helmet and magnetometer signals were recorded of the empty room for 60 s. The noise spectra were recorded at 1000 samples per second and are shown in Figure 5A. The spectra of the raw signals show the noise increase at higher frequencies, caused by the limited bandwidth of the sensors. This noise increase is also visible in the open-loop noise spectra when the noise is properly scaled with the response of the magnetometers. It is a general feature of OPMs due to their limited bandwidth, in this case of roughly 200 Hz. The same feature can be seen in cryogenic MEG, but at much higher frequencies due to the larger bandwidth on SQUIDs. In addition, the spectra are dominated by low-frequency noise and noise peaks around 10 Hz. The sources of the excess noise at lower frequencies include vibrations of the system in the room, noise from the panel coils, and noise from cryogenic MEG system. Signal-space projection (SSP) was applied to the data (Robinson, 1989; Uusitalo and Ilmoniemi, 1997). The spectra after applying 4 projections are shown in Figure 5B: much of the low-frequency noise was removed to a remaining average noise floor of 20 ± 5 fT/Hz^1/2^. While this could be improved in the future, it is sufficient for the current measurements in cleaning up the large amount of noise present in the room in the frequency band of interest.

### 4.2. Auditory data

A subset of ~100 OPMs was also tested on a set of healthy human adult subjects. The exact number varied slightly between subjects. Six healthy adults provided informed consent to participate in this study, which was approved by the Colorado Multiple Institutional Review Board. The subjects laid in a supine position on the bed with their head resting inside the helmet. Five head position indicator (HPI) coils were taped to the forehead, the nasion, and the left and right preauricular (LPA, RPA) of the subject for co-registration. A 3SPACE Polhemus magnetic 3D digitizer was used to co-register the locations of the HPI coils with respect to the fiducials as well as with reference points on the helmet. The subject’s head shape was also digitized relative to these fiducials. After the door of the MSR was closed, fields at the location of the sensors were zeroed by use of the automated procedure internal to the sensor system. This procedure uses a set of coils imbedded inside the sensor head. The sensor positions and orientations were determined with the automatic procedure described above.

Auditory-evoked fields were measured as a response to a short tone burst at 1000 Hz applied to the left ear with an interstimulus interval (ISI) of 4 s. The sounds were applied through a non-magnetic tube terminated in an ear foam insert. The sound level was chosen as 70 dB sound pressure level (SPL) above the hearing threshold of the subject. The duration of the tones was 100 ms with a 5 ms rise and fall time. The auditory delivery system had a delay of 34 ms between the trigger and the tone delivered to the earpiece, the data has not been corrected for that. E-Prime stimulus software (Psychology Software Tools) was used to deliver the tone and trigger. The trigger was collected in a simultaneous stimulus channel that is part of the integrated control system. Between 146 and 164 tones were presented to the subject over a 10-min span. The HPI coils were activated sequentially with a sinusoidal wave at 43 Hz for co-registration.

All subjects were tested under this paradigm consecutively with the OPM MEG HEDscan system described above and the cryogenic MEG system from 4-D Neuroimaging in a randomized order. The cryogenic system contains 248 axial first-order gradiometers. A sample rate of 1,000 Hz was used for the OPM MEG data collections and a sample rate of 678.17 Hz was used for the cryoMEG data collections. The data were collected with the widest bandwidth possible limited only by hardware antialiasing filters, if any.

#### 4.2.1. Evoked analysis

The OPM MEG and cryoMEG data underwent the same analysis pipeline using MNE python (Gramfort et al., 2013) and used the CTF/BTI head coordinate system convention. For each subject, the continuous data were filtered from 0.5–30 Hz and the 60-Hz power line was notched along with the 120 and 180-Hz harmonics. The data were epoched relative to the stimulus onset from-200 ms to 700 ms. The pre-stimulus baseline was then subtracted from each epoch. The epochs were visually examined for artifacts such as eye blinks and these epochs were removed. The remaining 148 epochs were averaged resulting in an evoked average of the auditory response. The N100 peak was identified for each subject and an overlay of all channels (butterfly) along with the topology at the N100 peak were plotted.

**Figure 6:**
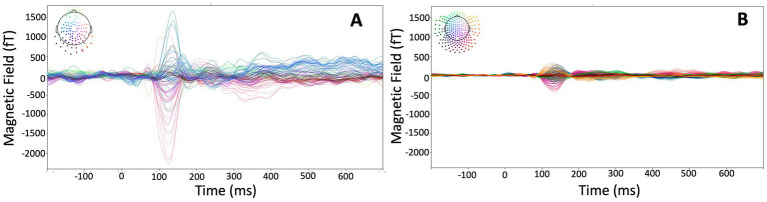
Auditory-evoked responses from the same subject measured with the HEDscan OPM MEG system [**(A)**: 148 averages, 100 channels] and 4-D cryogenic MEG system [**(B)**: 148 averages, 246 channels]. The colored pattern in the top left corners show the distribution of channels on the head, showing a dipolar pattern with blue signals having a positive, and red signals having a negative amplitude of the M100 signal.

**Figure 7:**
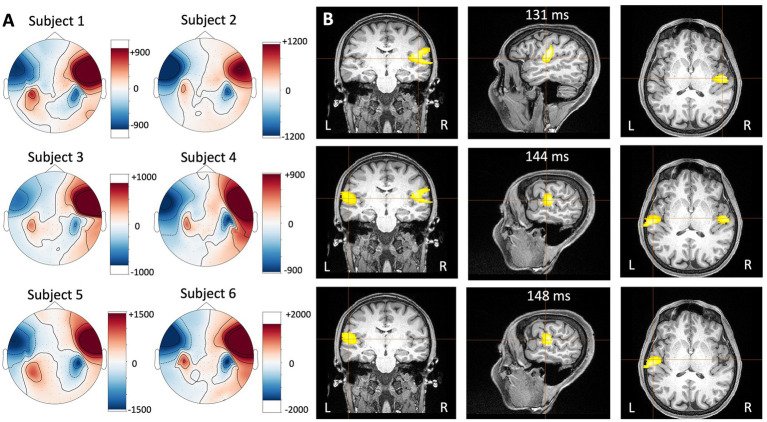
**(A)** Magnetic field distribution measured with the HEDscan system in six different subjects 123 ms – 135 ms post trigger to the left ear of the subject. Blue represents inward going fields and red represents outward going fields. **(B)** Event-related Beamformer results for subject 6 measured with the HEDscan system in response to sounds delivered to the left ear. It can be seen that the activity starts in the contralateral right auditory cortex (131 ms, top row), is followed by bilateral activity (144 ms, middle row), and finally activity appears in the ipsilateral left auditory cortex (148 ms, bottom row).

Auditory-evoked fields recorded with the HEDscan and 4-D system are shown in Figures 6A,B, respectively. Both recordings are displayed on the same scale, showing clearly the larger amplitudes recorded with the OPMs, but also the larger background noise. The magnetic field distribution 123 ms – 135 ms post trigger is shown for all six subjects in Figure 7A, showing bilateral dipolar patterns in the left and right temporal regions. The right-hand rule and the polarity of the field patterns indicate both dipoles are oriented away from the vertex (downwards pointing) consistent with cryoMEG findings.

#### 4.2.2. Beamformer analysis

The auditory-evoked responses were localized with the event-related beamformer in the Brainwave software (BWS) for all subjects. Since an MRI image was only available subject 6, template MRIs were used for the remaining subjects. The results were consistent across the individuals, i.e., the activity localized in a spot in the contralateral temporal region with timings consistent with the N100. The results for subject 6 are shown in Figure 7B as an example. The head shape points from the subjects were used to co-register the OPM MEG data and the subject’s MRI. The data was filtered from 1 to 30 Hz and the event-related beamformers were performed using 1 ms steps using a single sphere model. A best fit sphere was computed using the subjects’ head shape points. At 131 ms post trigger, the activity was localized to the contralateral right auditory cortex of subject 6 (Figure 7B top row), followed by bilateral activity at 144 ms (Figure 7B middle row), and activity in the ipsilateral left auditory cortex at 148 ms (Figure 7B bottom row). This spatio-temporal behavior is in agreement with literature results measured with cryogenic MEG systems (Näätänen and Picton, 1987).

## 5. Conclusion

In conclusion, we have described evaluation and validation measurements of the first full-head high-density 128-channel OPM MEG system. We have assessed important system parameters, such as linearity, response bandwidth, and crosstalk. Auditory-evoked responses have been measured in six healthy adults with a standard well-known paradigm using a subset of ~100 OPM sensors and 246 cryoMEG channels. Full-head OPM MEG systems are now available commercially and thorough testing and validation and understanding of these systems is essential.

## Data availability statement

The raw data supporting the conclusions of this article will be made available by the authors, without undue reservation.

## Ethics statement

The studies involving human participants were reviewed and approved by Colorado Multiple Institutional Review Board. The patients/participants provided their written informed consent to participate in this study.

## Author contributions

OA designed the OPM sensor. KHu oversaw the development of the control electronic system. SK oversaw the system integration. TM developed the sensor firmware. AG developed the interface software and programmed the control system. KHo developed the helmet. AP developed the sensor localization features and tested the sensor system. VL built the OPM sensors. CC performed the MEMS fabrication. MG made the active coil panels. EQ installed and optimized the coil panels. AS handled the subjects and operated the 4-D system. PT implemented the auditory paradigms. EK analyzed the 4-D data. IB oversaw the human subject testing. TC analyzed the OPM data. All authors contributed to the article and approved the submitted version.

## Funding

Funding was provided by the National Institute of Health under grants R44MH118154, R01NS094604, and R01EB019440.

## Conflict of interest

The authors declare that the research was conducted with a commercially available OPM MEG system developed by FieldLine Medical. OA, KHu, SK, TM, AG, AP, KHo, AP, VL, CC, and TC have developed this system and have a financial relationship to FieldLine Medical, which could be perceived as a potential conflict of interest.

The remaining authors declare that the research was conducted in the absence of any commercial or financial relationships that could be construed as a potential conflict of interest.

## Publisher’s note

All claims expressed in this article are solely those of the authors and do not necessarily represent those of their affiliated organizations, or those of the publisher, the editors and the reviewers. Any product that may be evaluated in this article, or claim that may be made by its manufacturer, is not guaranteed or endorsed by the publisher.
